# Towards Mixed-Initiative Human–Robot Interaction: Assessment of Discriminative Physiological and Behavioral Features for Performance Prediction

**DOI:** 10.3390/s20010296

**Published:** 2020-01-05

**Authors:** Caroline P. C. Chanel, Raphaëlle N. Roy, Frédéric Dehais, Nicolas Drougard

**Affiliations:** ISAE-SUPAERO, Université de Toulouse, 31400 Toulouse, France; raphaelle.roy@isae-supaero.fr (R.N.R.); frederic.dehais@isae-supaero.fr (F.D.); nicolas.drougard@isae-supaero.fr (N.D.)

**Keywords:** human–robot interaction, physiological computing, intelligent sensors, performance prediction, human behavior

## Abstract

The design of human–robot interactions is a key challenge to optimize operational performance. A promising approach is to consider mixed-initiative interactions in which the tasks and authority of each human and artificial agents are dynamically defined according to their current abilities. An important issue for the implementation of mixed-initiative systems is to monitor human performance to dynamically drive task allocation between human and artificial agents (i.e., robots). We, therefore, designed an experimental scenario involving missions whereby participants had to cooperate with a robot to fight fires while facing hazards. Two levels of robot automation (manual vs. autonomous) were randomly manipulated to assess their impact on the participants’ performance across missions. Cardiac activity, eye-tracking, and participants’ actions on the user interface were collected. The participants performed differently to an extent that we could identify high and low score mission groups that also exhibited different behavioral, cardiac and ocular patterns. More specifically, our findings indicated that the higher level of automation could be beneficial to low-scoring participants but detrimental to high-scoring ones, and vice versa. In addition, inter-subject single-trial classification results showed that the studied behavioral and physiological features were relevant to predict mission performance. The highest average balanced accuracy (74%) was reached using the features extracted from all input devices. These results suggest that an adaptive HRI driving system, that would aim at maximizing performance, would be capable of analyzing such physiological and behavior markers online to further change the level of automation when it is relevant for the mission purpose.

## 1. Introduction

In the near future, human and artificial agents will be deployed to handle several missions in different contexts working as teammates. Some examples of contexts for which mixed human–machine systems would be relevant are search and rescue missions [1]; dirty, dull, and dangerous (3D) environments [2]; Mars rovers [3]; and multi-UAVs operation [4,5].

On the one hand, robots can operate in hostile environments, can quickly compute actions, and reduce mission costs while maximizing production. On the other hand, humans can handle unexpected events, they have intuition, are creative and capable of making complex (e.g., ethical or moral) decisions. However, the human operator may experience high workload [6] and cognitive fatigue [7,8] that in turn can impair their attentional [9] and executive abilities [10]. In this cybernetic system context, several challenges have arisen such as the implementation of human–robots monitoring and supervision systems to optimize their interaction and adapt the role of the human operator [11] and the role of the artificial agents [4,12,13].

A promising avenue to deal with this issue is to consider Mixed-Initiative for Human–Robots Interaction (MI-HRI). This type of interaction has been defined by [14] as “A collaboration strategy for human–robot teams where humans and robots opportunistically seize (relinquish) initiative from (to) each other as a mission is being executed, […] the initiative is mixed only when each member is authorized to intervene and seize control of it”. More specifically, this approach aims at identifying which human or artificial agent is the most capable of achieving a given task considering the evolution of the mission (e.g., failure of the robot, cognitive fatigue or psychological stress). Technically, this presupposes to implement monitoring techniques to evaluate the states of the different artificial and human agents and to design a decision system to dynamically adapt their respective role and authority [12,15]. Whereas the state assessment of deterministic agents (i.e., robots) is relatively simple, the evaluation of the human mental states remains challenging. This latter point is critical as poor diagnostics can lead to inadequate adaptations of the overall system [16]. Indeed, triggering higher level of automation, as well as not triggering it when necessary, can dramatically degrade performance [17,18]. Thus, one can argue that it could be beneficial to identify relevant markers of human performance as long mixed-initiative systems are concerned.

To our knowledge, the literature is still scarce concerning the evaluation of human performance, in particular, for Mixed-Initiative Human–Robot Interaction (MI-HRI) systems [12,19,20]. Interestingly, some authors [11,21,22] demonstrated that behavioral metrics could be defined to assess human operators’ workload and performance when interacting with a varying number of robots. In the same vein, ref. [23] proposed a methodology based on time series clustering to identify operators’ profile during their training process by exploiting several performance metrics. Thereby, the use of eye-tracking data has also been shown to be a suitable technique to estimate the human operator’s state [24] and to dynamically reallocate tasks between the human and unmanned aerial vehicles [4]. Physiological features were explored in [25] to recognize affective states from human operators interacting with a robot, and in [26] to directly adapt the interface being used during the interaction. A relevant approach is also presented in [19], whereby physiological data collected from a galvanic skin device were used to estimate the user engagement during human–robot interaction.

The use of physiological features to predict the operator’s performance during MI-HRI appears to be a relevant approach. The work in [27] proposed a rich review on several behavioral and physiological markers well-proofread in different scenarios, being general, and promising for online computation, thus, particularly useful for MI-HRI. For instance, features derived from eye-tracking (ET) devices, such as the number and duration of fixations, blinks [28], and cardiac features collected with an electrocardiogram (ECG), such as Heart Rate (HR) and Heart-Rate Variability (HRV), have been shown to be useful for workload assessment in several experimental contexts [27,29] and/or cognitive engagement estimation [30], while being quite promising for online computing [31]. Both ECG and ET devices are noninvasive devices, and easy to use in real life settings, which make them highly relevant for human–machine teaming applications. Particularly, as ET provides the eye gaze position, it is highly relevant to measure the human operator eye gaze activity in the context of supervisory control and multi-tasking management [32].

To progress towards systems that would dynamically drive MI-HRI to enhance systems’ performance, a first step is to characterize reliable behavioral and psycho-physiological features that could account for human performance. For this purpose, one can exploit a real world like human–robot mission, in which the human interacts with an autonomous robot. We thus built an experimental protocol using the robotic mission of the Firefighter Robot Game (http://robot-isae.isae.fr) developed by [33] and exhaustively described in [34].

**Figure 1:**
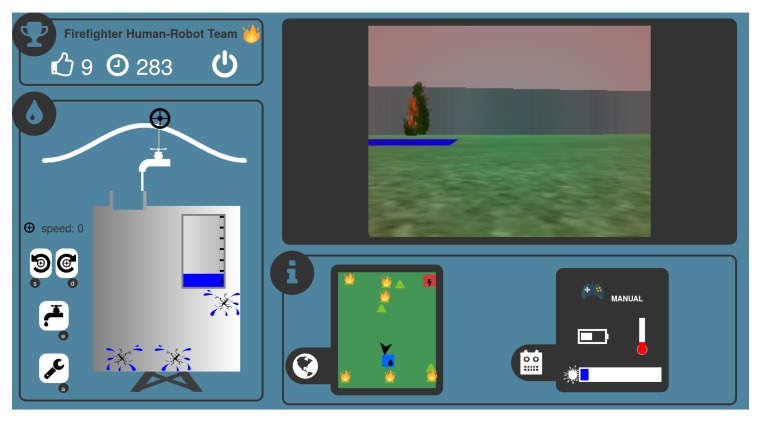
Screenshot of the graphical user interface (GUI) during a mission. **Top left**: remaining time and score. **Top right**: robot camera feedback. **Bottom left**: water stock management task. **Bottom right**: robot status.

This experimental set-up involved a user interface (see Figure 1) that allowed the participants to cooperate with a robot to fight fires. The robot could provide two levels of automation. In the high level of automation (i.e., autonomous robot mode) the robot navigates and extinguishes fires autonomously and the volunteers have to supervise the robot’s status and manage the water tank supply level. In the low level of automation (i.e., manual control), the participants have to perform the task manually such as handling the robot, extinguishing fires and managing the water tank supply level. The level of automation was randomly changed during the mission every ten seconds so as to assess its impact on the overall performance of the human–robot system (i.e., the number of extinguished fires). Different behavioral and psycho-physiological measures were collected during the experiment. First, participants’ actions (keystrokes and mouse clicks) on the user interface were monitored. An eye tracker (ET) was also used to measure the number of fixations on the main areas of interest (AOIs), and an electrocardiogram allowed to derive the Heart Rate (HR) and Heart-Rate Variability (HRV).

Thus, the objective of this study was to relate these different behavioral and psycho-physiological metrics to the mission performance. We used a two-step approach to demonstrate that participants’ behavioral, ECG and ET features would allow to discriminate between high and low mission-performer given the level of automation of the robot (manual control versus autonomous mode). First, we conducted statistical analysis for each of the dependent variables at the group level. Second, we implemented inter-subject single-trial classification algorithms that combine these different features to predict mission performance.

## 2. Materials and Methods

### 2.1. Participants

A total of 18 participants (5 females; mean age: 27.88; sd.: 4.76), all students from the ISAE-SUPAERO engineering school, were recruited by means of electronic mail announcements and oral advertising. All participants had normal (or corrected) vision abilities, and subscribed to social security and civil responsibility insurances. They all gave their informed consent. The project was validated by the local ethical committee of the University of Toulouse (CERNI number 2018-070).

### 2.2. Firefighter Robot Mission Task

This human–robot mission immerses the human operator in a scenario where s/he plays the role of a remote fireman who has to cooperate with a robot that is evolving in a virtual environment composed with trees that can randomly self-ignite. The goal of the mission is to fight as many fires as possible in a limited amount of time. Through the graphical user interface (GUI) shown in Figure 1, the human operator gets the video streaming from the robot’s camera (top right) as well as the position of the robot in the map (bottom center) and s/he monitors the robot’s internal states, namely, the temperature, the automation level, the battery charge level, and the amount of water in the on-board tank (bottom right). The human operator also has to manage an external water tank (bottom left) during all the mission using the buttons on the left-side of the interface: a tap, which can move horizontally by controlling a wheel (top buttons), fills the external tank when it is in the middle (which is an unstable equilibrium). To actually fill this external tank, the button bellow (black tap) turns on the tap for few seconds. Moreover the participants have to fix water leaks that may randomly occur on this external water tank by using the last button (black wrench).

The amount of water (i.e., the water in the robot on-board tank) that can be expulsed from the robot to put out fires is not unlimited: the robot has to be in the vicinity of a water filling station represented by a blue square on the ground to refill its on-board tank. The water of the filling station comes from the external water tank that must therefore not be empty. Despite the difficulty in filling this external tank and the occurrence of leaks, the human operator has to ensure that enough water will be available to the robot. In addition, the battery charge level of the robot decreases over time. It can be recharged at a charging station represented by a red square in the environment. If the battery is empty and the robot is not on the red square, the mission fails and ends prematurely. The temperature of the robot increases when it is too close to the flames and, it can also end the mission if too high.

In autonomous operation mode, the robot is able to navigate autonomously, and is programmed to extinguish the nearest fire, or to refill water or to recharge battery when necessary. This mode relates to a high automation condition, where the human operator has to supervise the robot’s parameters, and to manage the filling and repairing of the external tank. The robot’s manual control relates to a low automation level condition, because participants have to perform additional tasks, such as manually controlling the robot, managing its resources, and extinguishing fires. In this low automation level, the robot needs to be controlled by the keyboard arrows (navigation), and the participant has to press the keyboard space bar to shoot water. Note that, for the aim of this study, the robot’s automation level is changed randomly every ten seconds during the mission, following a uniform probability distribution over automation levels. In other words, every 10 s there was 50% of chance that automation level changes or remains the same. As we study the impact of the robot automation level, a random change of the automation level is the best choice to avoid to create a bias related to a mode change policy; 10-s time windows were chosen as data collected during pilot experiments, as well as previous study [29], demonstrated to compute HR and HRV metrics in a robust manner for online purpose.

Each mission lasted at most 10 min. This design ensures a compromise between (i) the number of 10-s time windows for further statistical analysis and classification purpose. 60 × 10-s time windows per mission were expected, resulting on around 30 expected episodes of 10 s for each automation level condition. However, in our robotic scenario some missions could prematurely abort (robot battery fully discharged for instance), thus the average number of 10-s condition windows would be a little lower than 30; (ii) to induce time pressure for engaging participants (a countdown timer was displayed, see Figure 1 top-left) while avoiding fatigue issues given that the participants had to perform several missions. The complete experimental protocol is described in the following section.

### 2.3. Experimental Protocol

Participants were first welcomed in the experimental facilities at ISAE-SUPAERO. After reading and giving their signed consent, a training mission was proposed during which no data were recorded. The next step was to install the ECG device and to proceed with the calibration of the eye-tracking system. Once data acquisition was carefully checked, the participants had to fill the Karolinska Sleepiness Scale (KSS) survey [35] (see the paragraph “Subjective Feedback” Section 2.5.1). Finally, the participants had to perform four missions, each of them following the protocol below.

1. Rest period: the participants focused on a black cross in the center of the screen during 1 min. This allows to obtain a cardiac baseline.
2. Mission: the mission is launched and its maximum duration is 10 min.
3. Questionnaire: when the mission is finished, the participants are asked to fill the NASA-TLX survey [36] to get their subjective feedback concerning the effort made to carry out the mission (see the paragraph “Subjective Feedback” Section 2.5.1).
4. Break: a 2 min break is proposed to the participants.

**Figure 2:**
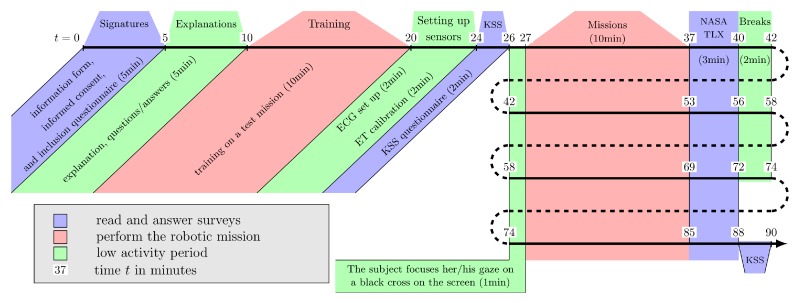
Experimental protocol time line.

After the end of the four missions, the participants are asked to fill again the KSS survey. The experiment was finalized by checking data records and removing the ECG equipment from the participant. The experimental protocol is summarized in Figure 2. As the participants have to perform four missions, the experiment lasted about 1h30 including training, device set up, surveys, and scenarios.

### 2.4. Data Collection

All robotic and mission state variables, as well as human operators’ actions on the ground station (keyboard inputs and mouse clicks), eye-tracking, and ECG data were recorded using Lab Streaming Layer (LSL) (https://github.com/sccn/labstreaminglayer). LSL is a middleware developed by the Swartz Center for Computational Neuroscience (UCSD), for unified collection of experimental measurements. This framework allows time-synchronization and online access to data while avoiding networking issues. LSL also provides a software, called LabRecorder, that allows to select and record all data of interest.

The robot position, battery level, amount of water available on board, temperature and automation level as well as the amount of water in the external tank, the state of the forest, elapsed time, leak position and participant actions on the interface were streamed via LSL each second [34]. With regard to the ECG equipment, data stream software was developed by Mega Electronics for LSL (software available at https://github.com/bwrc/faros-streamer). Faros ECG raw data (500 Hz) and Inter-Beat Interval (IBI)—or RR peaks intervals—were recorded. A Python script was developed based on the SMI SDK and LSL to produce streams containing the SMI measured gaze and fixation events.

### 2.5. Data Analysis

#### 2.5.1. Data Processing

##### Performance Scores

The global mission performance was assessed using the total number of extinguished fires (see Section 2.2). Note that the human operator must properly manage the filling of the external tank, monitor the robot’s parameters (water reserve, battery, temperature, and automation level) and ensure manual control when necessary in order to reach a high mission score. In summary, the global mission score is intrinsically dependent on all of these tasks.

However, to evaluate the impact of the robot’s automation level, we also computed the total number of extinguished fires effectively done by the robot in autonomous mode and the total number of extinguished fires effectively performed by the human operator in manual control. In other words, for a given mission and a given automation level, the total number of fires is computed as the sum of fires extinguished during all 10-s time windows.

##### Subjective Feedback

The subjective feedback is composed by the scores collected with the NASA Task Load Index (or in short NASA-TLX) survey [36] and the Karolinska sleepiness scale (or KSS) questionnaire [35]. The NASA-TLX is a survey meant to assess the subjective task workload. It computes an overall workload score based on a weighted average of ratings of six dimensions: mental demand, physical demand, temporal demand, performance, effort, and frustration. The html version (available at https://www.keithv.com/software/nasatlx/), which automatically computes the score, was used in our experiment. Note that a high NASA-TLX score is associated with a high subjective workload. The KSS is a survey that is generally used to measure the subjective sleepiness (i.e., fatigue). The 9-graded KSS was used in our experiment. A high KSS score can be associated with a significant drowsiness.

##### Behavioral Data

The behavioral data concerns the human operator’s actions (keystrokes and mouse clicks) on the interface recorded every second. Only keystrokes and mouse clicks related to the different sub-tasks were considered (e.g., typing error was filtered). Thus, to assess on which tasks the participant’s actions were focused, we were interested in the average number of keystrokes/clicks performed and related with navigation and external tank sub-tasks given the robot’s level of automation, mission, and participant.

Additional metrics could also be computed based on the behavioral data. As stated before, the global mission score depends on a correct water level management of the external tank because the robot needs to recharge in water from it to extinguish fires. Therefore, given the fact that this sub-task needs to be performed by the human operator during the whole mission, we are also interested in the average variation of the water level of the external tank given the robot’s automation level, mission and participant. Since the time period between two automation level changes is a multiple of 10 s, the water level variations v∈R of the external tank are defined on successive (nonoverlapping) 10 s windows as the difference between the level at the end of the window $l_{e}\in R$ and the level at the beginning $l_{b}\in R$: $v=l_{e}-l_{b}$.

##### Eye-Tracking

**Figure 3:**
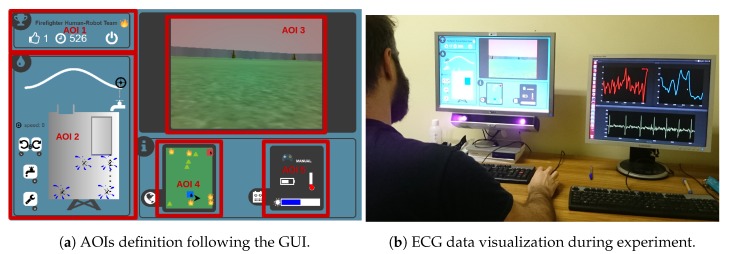
(**a**) Screenshot of the graphical user interface showing the areas of interest (AOIs) defined for the study; (**b**) ECG-data acquisition during the experiment.

Gaze movements and fixations events were recorded using the SMI Red 250 Hz ET system. We set a temporal threshold to 80 ms to identify instream fixation events. Five areas of interests (AOI, see Figure 3a) were defined on a reference image: AOI 1 refers to the current mission score and remaining time information; AOI 2 refers to the external tank task; AOI 3 represents the robot video stream; AOI 4 is the map of the zone; and, the AOI 5 summarizes the robot’s state variables related to the battery, the temperature, the amount of water, and the robot’s automation level. The Eye-Tracking device automatically detects fixations, i.e., when the gaze fixes more than 80 ms on a given screen point, and returns gaze location for each of them. Let us denote by $f_{n}$ the timestamp of the *n*th fixation and by $pos_{n}=\left(x_{n},y_{n}\right)\in R^{2}$ its position on the screen. Given the time window $w=[w_{a},w_{b}]$ where $\left(w_{a},w_{b}\right)\in R^{2}$ with $w_{a}<w_{b}$, we can define the set of considered indices $W=\{n\in N|f_{n}\in w\}\subset N$.

Now, given one of the screen zones (AOIs) shown in Figure 3a, here denoted by $AOI_{i}$, the total number of fixations in $AOI_{i}$ is computed as follows,

(1) $$FIX_{i}\left(W\right)=\sum_{n\in W}{𝟙}_{\left\{pos_{n}\in AOI_{i}\right\}},$$

where i∈{1,…,5} and ${𝟙}_{A}=1$ if *A* is true and 0 otherwise. This total number of fixations in each AOI $FIX_{i}$ is computed in this study on the 10-s time windows given the robot’s automation level, mission, and participant.

##### ECG Data

An ECG was used to collect participants’ cardiac activity at a sampling rate of 500Hz with the Faros Emotion 360 system. This ECG device filters the data and automatically extracts and streams the inter-beat intervals (IBI) or R-R intervals. *Heart Rate* (HR) was computed in beats per minute (bpm) using the serie of R-R intervals [37,38]. Given that $IBI_{n}=R_{n}-R_{n-1}$, with $R_{n}$ as the timestamp of the *n*th R peak (i.e., highest positive peak), and given time window $w=[w_{a},w_{b}]$ with $\left(w_{a},w_{b}\right)\in R^{2}$ with $w_{a}<w_{b}$, we can define the set of considered indices $W=\{n\in N|R_{n-1}\in wandR_{n}\in w\}\subset N$.

Thus, the mean HR can then be computed as follows,

(2) $$HR\left(W\right)=\frac{60\cdot \#W}{\sum_{n\in W}IBI_{n}},$$

where, #W states for the cardinal of the set W.

The *Heart Rate Variability* (HRV) can also be computed in the time domain as the variability of the Inter-Beat Intervals (IBI):

(3) $$HRV\left(W\right)=\sqrt{\frac{1}{\#W-1}\sum_{n\in W}{\left(IBI_{n}-\frac{60}{HR(W)}\right)}^{2}}.$$

Note that data collected during the rest periods (one minute before each mission, see Section 2.3) is used as a baseline for normalizing HR and HRV temporal metrics (computed with the Equations (2) and (3)) for a given mission and participant. Thus, for a given mission and participant, the set of indices associated with the rest sessions are denoted by $W_{rest}$, and the normalized HR and HRV are computed as follows,

(4) $$\begin{array}{ccc} HR_{norm}\left(W\right) & = & HR\left(W\right)-HR\left(W_{rest}\right), \end{array}$$

(5) $$\begin{array}{ccc} HRV_{norm}\left(W\right) & = & HRV\left(W\right)-HRV\left(W_{rest}\right). \end{array}$$

Thanks to this normalization, the ECG-based metrics of each mission performed by the 18 participants become inter-participant comparable physiological data. Thus, based on the Equations (4) and (5), we computed (i) the normalized HR ($HR_{norm}$) and the normalized HRV ($HRV_{norm}$) for the overall mission (10-min time window) per participant and mission; and (ii) the average of normalized HRs and HRVs computed on 10-s time windows, given the robot’s automation level, the mission, and the volunteer.

#### 2.5.2. Statistical Analysis

Given the noncompliance of the normality assumption for all the computed metrics, all statistical analyses were ran using nonparametric tests from R packages v3.4 (available at https://cran.r-project.org/).

##### Mission Performance Analysis

A Spearman rank correlation was computed between the NASA-TLX and the mission scores (i.e., total number of extinguished fires). A Spearman rank correlation matrix was also computed between the physiological markers (i.e., ECG-based metrics), and the total mission score and the NASA-TLX score.

##### Behavioral and Physiological Markers’ Analysis Depending on the Robot Automation Level and the Performance Group

The main objective of this study is to assess whether the participants’ physiological and behavioral markers are impacted by the automation level in a different way if they perform the mission well or poorly. It was possible to characterize, using the median score of the 72 missions (4 missions × 18 participants), two well-balanced groups of mission performance: high score and low score groups. Such an analysis is based on the actual participant’s performance for a given mission, rather than average individual’s performance over all missions.

Thus, a Wilcoxon test was applied to compare the difference between the markers from the two groups (i.e., high and low score groups). However, when considering performance group and automation level, a pairwise Wilcoxon test for multiple comparisons with *p*-value adjustement was performed.

Moreover, nonparametric tests for multivariate data and many factor levels [39] were applied to behavioral (i.e., keystrokes/clicks) and eye-tracking (i.e., number of fixations on AOIs) markers. The factor levels concern the interaction between robot’s automation level and performance group. This test was chosen because the average number of actions on the interface (keystrokes/clicks) related to each sub-task, as well as, the average number of fixations in different AOIs, are dependent variables. Note that a pairwise Wilcoxon test for multiple comparisons with *p*-value was applied between factors levels as a post hoc test. These post-hoc tests were applied only to the dependent variables for which the Krushkal–Wallis test presented significant results.

#### 2.5.3. Classification

Finally, two-dimensional classifiers were built to evaluate the performance predictive power of the behavioral and physiological features. More precisely, inter-subject single-trial classification models were studied exploiting the dataset composed by the ECG, the ET, and the keystrokes/clicks features, computed for 10-s time windows of a given automation level (see the paragraphs “Behavioral Data”, “Eye-tracking” and “ECG data” respectively in Section 2.5.1). Each sample was labeled by the related mission performance (high or low score). The total dataset is composed by 3591 samples, with 1459 samples for the low-scoring group and 2132 for the high-scoring one. Note that this unbalanced dataset can be explained by the fact that some low-scoring missions ended prematurely. In addition, several combinations of features were also studied in order to evaluate their contribution on the performance prediction with respect to the input device. The different combinations evaluated consisted in arrangements of ECG (i.e., HR and HRV), ET (i.e., number of fixation on each AOI), keystrokes/clicks (i.e., the number of keyboard inputs and clicks related the external tank and robot navigation) input features, all conditioned by the automation level of the robot. Thus, the automation level of the robot constitutes an additional common input feature for all tests. For instance, when ECG features are leaved out, classifications models were trained with only ET, keystrokes/clicks and the automation level of the robot as input features.

Well-known classifiers, such as, k-Nearest Neighbors (kNN), Linear and Quadratic Discriminant Analyses (LDA, QDA), Support Vector Machine (SVM), Gaussian Process (GP), Decision Trees (DT), Random Forest (RF), Neural Network (NN), AdaBoost (ADA), and Naive Bayes (NB), from the scikit-learn library version 0.20.3 were used. A grid search algorithm was applied for hyper-parameters optimization using 80% of data samples for all concerned algorithms given the balanced accuracy score. Then, each classifier was 5-fold cross-validated (CV =5) twenty times (20×5-fold); based on these runs, the average balanced accuracy and the 95% confidence interval were computed.

## 3. Results

### 3.1. Subjective Feedback

**Figure 4:**
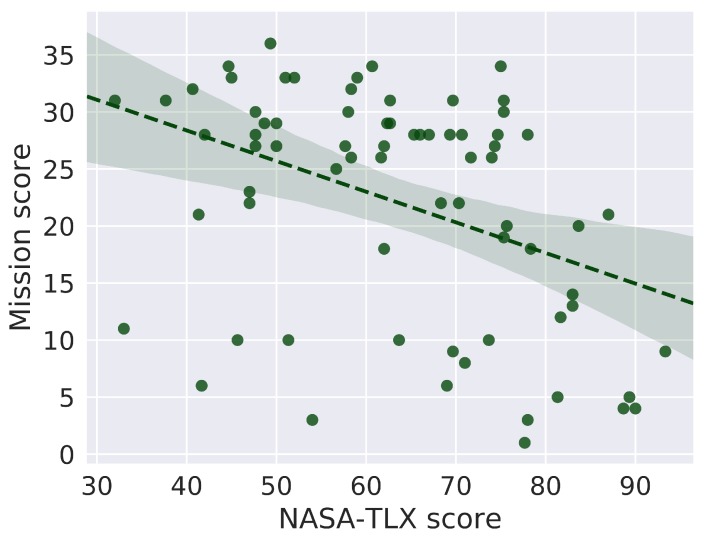
Final mission score versus NASA-TLX score. Each plotted point relates to one of the 72 missions.

The Spearman’s rank correlation disclosed a significant negative correlation between the NASA-TLX and the global mission score (ρ=−0.42,N=72,p<0.001). Figure 4 plots the final mission score versus the NASA-TLX score for each participant and mission.

A paired Wilcoxon test revealed no statistical difference between the KSS score between the beginning and the end of the experiment.

### 3.2. Behavioral Markers

#### 3.2.1. Operator’s Contribution to Mission Score Given the Performance Group

**Figure 5:**
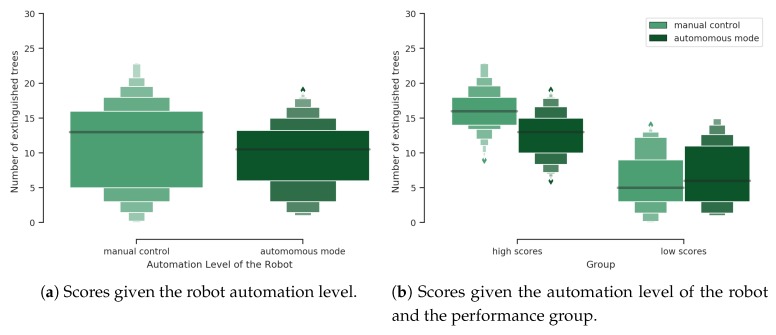
Score analysis given the automation level of the robot and the performance group.

Note that the robot is able to shoot water alone when in autonomous mode. Thus the actual operator’s score contribution relates to the number of fires extinguished when the robot is manually controlled. Figure 5a shows the scores reached considering all missions given the robot automation level. A Wilcoxon test showed that manual control scores are significantly higher then scores in autonomous mode (p<0.05) regardless of the performance group. However, a deeper analysis performed to evaluate the total number of extinguished fires depending on the robot automation level and performance group brought different results. A pairwise Wilcoxon test for multiple comparisons with p-adjustment showed that when the mission is part of the high score mission group, the participants have extinguished more fires in manual control than the robot in autonomous mode (p<0.001). As expected, during these high score missions, more fires were extinguished than during the low score missions, either in manual control (p<0.001) or in autonomous mode (p<0.001). Interestingly, for the low score group, the robot extinguished more fires in autonomous mode (p=0.05) than when the human operator was manually controlling the robot (see Figure 5b).

#### 3.2.2. Operator’s Actions on the Interface Given the Robot Automation Level and the Performance Group

##### External Tank Water Level Variation With Respect to the Performance Group

A pairwise Wilcoxon test for multiple comparisons with p-adjustment showed that the high-scoring group reached a higher average variation on the external tank water level than the low-scoring group in autonomous mode (p<0.001). As expected, both the low score group (p<0.001) and high score group (p<0.001) presented a positive variation on tank level during the autonomous mode compared to manual control.

##### Keystrokes/Clicks Given the Robot Automation Level and Performance Group

**Figure 6:**
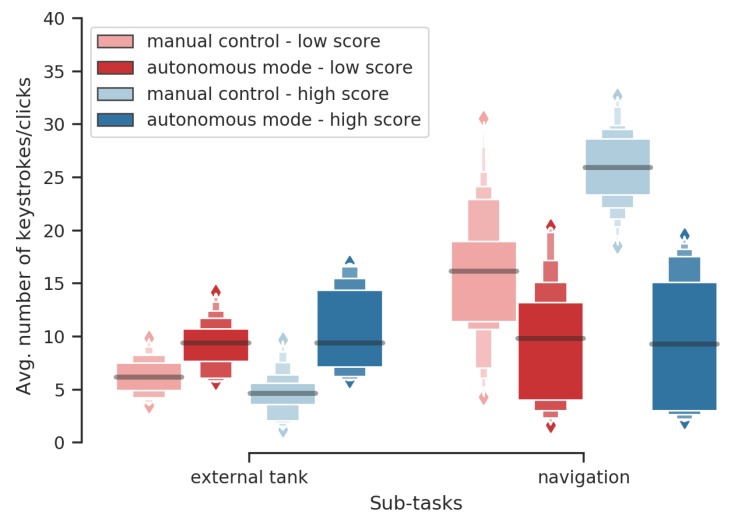
Average number of keystrokes/clicks per sub-task (filling the external tank and driving the robot) during autonomous mode and manual control given performance groups. These graphical representations reappear in the next figures. They show a large number of quantiles, which makes it easier to visualize nonparametric distributions.

A nonparametric test for multivariate data [39] was performed for the averaged number of keystrokes/clicks related to the external tank and navigation task. The four factor levels considered are the combinations of the two performance groups and the two levels of automation. A significant effect of this interaction was observed. The McKeon approximation for the Lawley Hotelling test gives a statistics of F(6.000,183.5663)=105.336, with p<0.001. These results are illustrated by Figure 6.

Post-hoc tests showed that

- when the robot is in autonomous mode, participants use more actions (keystrokes/clicks) related to the external tank during high score missions than during low score missions (p=0.01);
- when the robot is in manual control, participants use more the navigation-related keystrokes during high-score missions than during low score missions (p<0.001); and
- in autonomous mode participants use more the navigation-related keystrokes during low score missions than high score missions (p<0.01). Note that it represents behavioral errors since the robot does not need to be driven in autonomous mode (high automation level).

### 3.3. Eye-Tracking Markers

**Figure 7:**
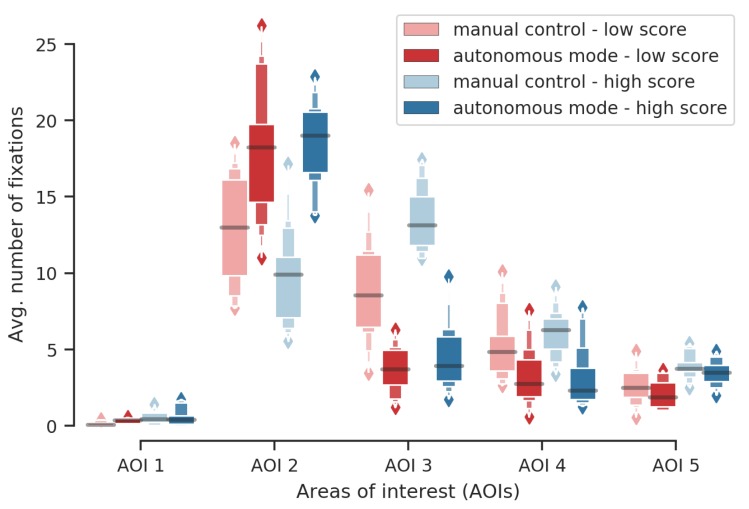
Average number of fixations on AOIs during autonomous mode and manual control, given the performance group. AOI 1: remaining time; AOI 2: external tank; AOI 3: robot video stream; AOI 4: map; and AOI 5: robot’s parameters.

The same nonparametric test for multivariate data was performed for the number of fixations on the different AOIs. A significant effect of this interaction was observed (McKeon approximation for the Lawley Hotelling test: F(15,251.7024)=34.694,p<0.001). These results are illustrated by Figure 7. Post hoc tests applied to relative effects between participants’ group and the robot automation level showed that

- participants have more fixated AOI 1 (chronometer and score) during high score missions in both modes (with p<0.001 for autonomous, and p<0.001 for manual) than during low score missions;
- participants have more fixated AOI 3 (robot video stream) during high score missions in both modes (autonomous p=0.01, manual p<0.001) than during low score missions;
- participants have more fixated AOI 4 (robot status) during high score missions in both modes (p<0.001 for both) than during low score missions.
- participants have more fixated AOI 2 in autonomous mode (p<0.05) during high score missions than during low score missions; and
- participants have more fixated AOI 2 in manual control during low score missions (p<0.001) than during high score missions. Note that it possibly demonstrates that these participants kept paying attention to the external tank sub-task instead of taking the robot’s manual control.

### 3.4. ECG Markers

#### 3.4.1. ECG Markers, Mission Performance and NASA-TLX Score

A Spearman’s rank correlation matrix with adjusted *p*-value was computed between the table of normalized HR and HRV markers ($HR_{norm}$ and $HRV_{norm}$, cf. Equations (4) and (5)), and the table of mission and NASA-TLX scores. Note that the time window used here is the total duration of the mission (the associated set of R peak indices is denoted by W).

Results showed that $HRV_{norm}\left(W\right)$ is correlated with NASA-TLX scores (ρ=0.42, p=0.001). However, only trends were observed between $HRV_{norm}\left(W\right)$ and mission score (ρ=−0.24, p=0.07), and between $HR_{norm}\left(W\right)$ and mission score (ρ=0.27, p=0.06).

#### 3.4.2. ECG Markers’ Analysis with Respect to Performance Group

**Figure 8:**
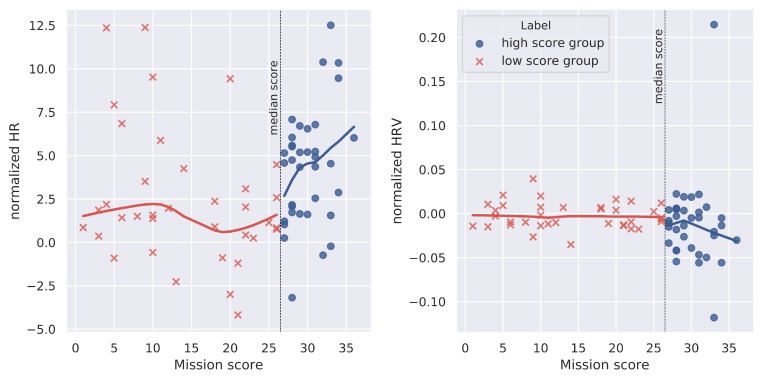
Normalized HR and HRV markers by performance group plotted in function of the final mission score. Each plotted point relates to one of the 72 missions.

A Wilcoxon test showed that participants have a significantly lower $HRV_{norm}\left(W\right)$ during high score mission than during low score missions (W=474, p<0.05). Also, $HR_{norm}\left(W\right)$ of the participants during high score missions is significantly higher than $HR_{norm}\left(W\right)$ during low score missions (W=883, p<0.01). These markers are illustrated in Figure 8 in function of the mission score.

#### 3.4.3. ECG Markers’ Analysis Depending on the Performance Group and the Robot Automation Level

**Figure 9:**
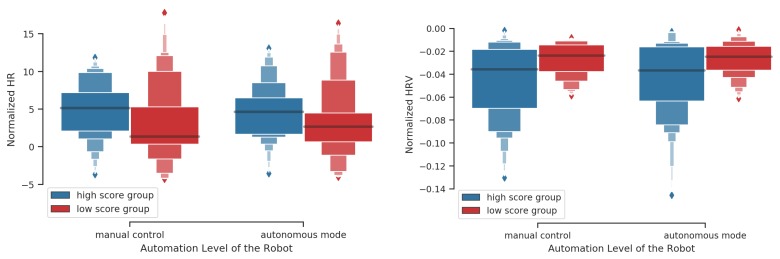
ECG features during autonomous mode and manual control given participants’ performance group.

A nonparametric test for multivariate data was performed on averaged ECG features ($HR_{norm}$ and $HRV_{norm}$) computed on 10-s time windows given the robot’s automation level and performance group. A significant effect was found (McKeon approximation for the Lawley Hotelling: F(6,183.5)=2.323,p<0.05). Post hoc tests show a trend for $HRV_{norm}$ (p=0.09 for both automation levels) and $HR_{norm}$ (p=0.07 for both automation levels): the high score group presents a lower $HRV_{norm}$ and a higher $HR_{norm}$ compared to the low score group regardless of the robot’s automation level (cf. Section 3.1; Figure 9).

### 3.5. Classification

**Figure 10:**
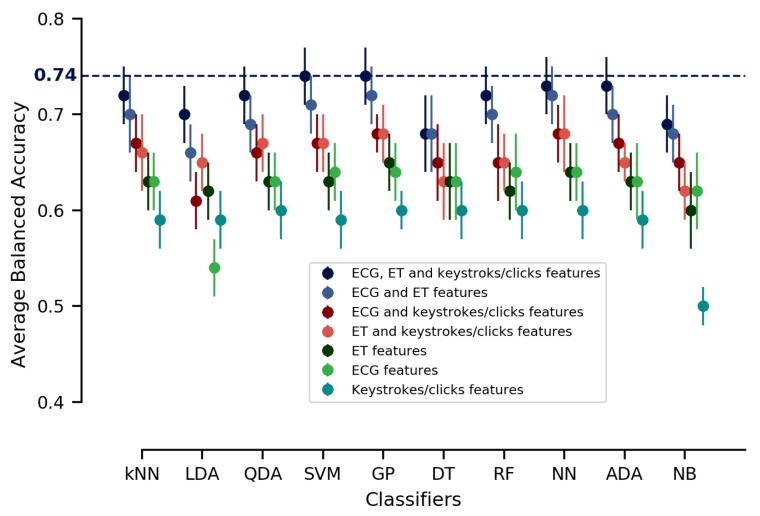
Average balanced accuracy for 20×5-fold cross-validations and the 95% confidence interval. Input features were conditioned on the automation level of the robot, which constitutes an additional common input feature for all tests. kNN: k-Nearest Neighbors; LDA: Linear Discriminant Analyses; QDA: Quadratic Discriminant Analyses; SVM: Support Vector Machine; GP: Gaussian Process; DT: Decision Trees; RF: Random Forest; NN: Neural Network; ADA: AdaBoost; and NB: Naive Bayes.

The average balanced accuracy and the 95% confidence interval results are illustrated in Figure 10 and summarized in Table 1. Interestingly, the best results found for all classifiers were obtained when all features—ECG, ET and keystrokes/clicks all conditioned on the automation level—were used as input features. The balanced accuracy results ranged from (0.74±0.03) to (0.68±0.04). When keystrokes/clicks features were removed, the results ranged from (0.72±0.03) to (0.66±0.03). When ET features were removed we obtained balanced accuracy results between (0.68±0.02) and (0.61±0.03). Balanced accuracy results ranged between (0.68±0.03) and (0.62±0.03) when ECG features were removed. Finally, when each ECG, ET or keystrokes/clicks features conditioned on the automation level of the robot were separately considered, the results were much less attractive.

The results indicated that the different classifiers (LDA, SVM, etc.) disclosed similar performance accuracy for a given set of input features (please report to mean and confidence interval in Table 1 and Figure 10). The highest average balanced accuracy was reached when all features were used as input features in this inter-subject single-trial approach, reflecting feature importance. These results demonstrated that all features were relevant to be used together for performance prediction. If we were to use a particular classifier, we would prefer the classifiers that have the best prediction results, i.e., here Support Vector Machine (SVM) and Gaussian Processes (GP). If it was necessary to train new classifiers several times, it might be better to use GP which has a lower computation time while a good prediction accuracy.

## 4. Discussion

The main objective of this study was to identify behavioral and psycho-physiological markers underpinning human’s performance that could be further used to drive the level of automation of robots in a mixed-initiative human–robot interaction (MI-HRI) paradigm. To assess discriminative physiological and behavioral features potentially relevant for MI-HRI, we designed an experimental scenario whereby the volunteers had to cooperate with a robot to fight forest fires. The level of automation of the robot (manual control versus autonomous mode) was randomly changed so as to measure its impact on the global performance of the human–robot system.

As previous works demonstrated that even experts may exhibit different abilities to perform similar tasks [40], we observed a variability among mission scores (as illustred in Figure 4). This observation is the cause of our choice in defining two mission score groups, separated by the median score (see Figure 8) that ensures two balanced groups. In our experiment, the high score group fought more fires as a consequence of a better navigation with the robot and better ability to manage the water tank in a given mission. Consistently, these two groups characterized by their mission score also differed at the psycho-physiological level. In the high score group, participants exhibited higher HR and lower HRV than in low score ones. Such an increase of HR and decrease of HRV are known to reflect a higher influence of the orthosympathetic nervous system on the autonomous nervous system, thus suggesting an increased catabolic activity to support the mobilization of cerebral resources to face the situation [41]. Conversely, poor behavioral performance associated with higher HRV may reveal task disengagement, and consequently an inability to face multitasking demands [42].

The distinction between high and low score missions allowed to conduct further analyses to assess the impact of the level of automation on the respective participants’ performance. This analysis disclosed that in the low score mission group, the autonomous mode of the robot improved the performance of the participants (i.e., more extinguished fires) whereas it had the opposite effect on the participants during the high score missions (see results in Section 3.2.1 and Figure 5b). Indeed, in the latter group, the participants fought more fires when manually controlling the robots. Moreover, this experiment revealed that the interaction with the robot could lead to conflict with automation. For instance, in low score missions, the participants experienced poor situation awareness of the current level of automation of the robot (see Section 3.3 concerning ET results), a phenomenon known as “mode error” [43] that led them to exhibit typical perseverative behaviors [10]. Indeed, during these low score missions, the participants erroneously attempted to manually control the robot in autonomous mode as revealed by their navigation related keystrokes. Conversely they also failed to take over when necessary as they hit less the navigation related keys when the robot was in manual control (low level of automation) than the participants during missions of the high score group. A reasonable explanation is that they were particularly focused on managing the external tank as revealed by a higher fixation rate on the external tank related AOI. Additionally, they glanced less at the robot’s parameters related AOI, therefore increasing the risk of ignoring important events (e.g., critical temperature or battery level) leading to abort the mission (cf. Section 3.3).

The statistical analysis allowed to assess promising physiological and behavioral features to predict performance depending on the robot automation level. Based on these results, ECG, ET and behavioral features were then used to train several classification algorithms in an inter-subject single-trial approach. Classification results showed that the highest average balanced accuracy was reached when all features were used together (see Figure 10) independently of the used classification algorithm. These results demonstrated the predictive power of these features. Recent works in robot teleoperation contexts have studied the use of ECG and/or ET features [24,44] to monitor human operator mental workload level or to detect degraded mental states. Thereby, and differently from them, our aim in this study was to highlight promising performance features that would help the HRI driving system to enhance human–robot interaction. In this sense, note that such a performance prediction would then be exploited by an intelligent HRI driving system to chose (or not) to change the automation level of the robot given the expected long-term performance.

### 4.1. Conclusion and Limitations

This paper is a first step towards MI-HRI driving systems. One first limitation is that we only manipulated two levels of automation. Such an approach already represented a challenging problem and proposed interesting results concerning useful discriminative features to predict mission performance. To go further, an interesting point would be to consider more levels of automation and their impact on the physiological and on the behavioral performance features. Sliding autonomy [45] literature gives examples where human operators could deliberatly choose the control mode [46], could be solicited to identify targets [4,5], or indicate to the system their intention of taking manual control [34,47]. Interestingly, the authors of [48] studied the impact on the human operator performance considering three levels of automation in a multi-robot scenario. They showed that a moderate automation level improved participants’ performance, and that higher levels of automation induced task disengagement. However, they also suggested that, due to individual differences that were quantified with an eye-tracking device, the appropriate automation level should depend on the underlying characteristics of the operator. Our results also go in this direction since they show that the automation level should be chosen in order to favor the upcoming mission performance given the human operator’s features pattern for instance.

This study has also other limitations related to the complexity of the tasks and the number of measures that were collected and analyzed (e.g., eye tracking, physiological, and subjective measures). It thus limits our interpretation and the ability to clearly isolate the relationship between the degree of autonomy of the robot and all the considered measures that account for our participants physiological and behavioral performance. This is an important issue as long as the sample size of volunteers was rather small (n= 18) and homogeneous (i.e., same level of education and young people) that were not totally representative of the population of operators interacting with robots. Moreover, it is also recommended that gender be better balanced in our sample of participants. We should therefore conduct experiments with a larger sample of participants with different levels of studies and with different ages especially as long as several variables (e.g., age, gender, and body mass index) may affect physiological and behavioral response.

Additionally, in MI-HRI systems, which is the context of this work, initiative is mixed only when any agent (human or robot) is able to seize the initiative from the other [14,47]. It means that, in the MI context, allowing the operator to choose the control mode should only be possible if the interaction driving system considers it beneficial for the mission. For instance, consider an event that can only be handled by the human operator, and assume that the operator did not notice it. The interaction driving system would switch the robot control mode to autonomous mode, while informing the human operator that a significant event has occurred, even if the human operator intends to perform manual control. In another context, the operator’s intention to take control of the robot can be used by the interaction driving system to pass control to the operator to optimize the operation.

### 4.2. Practical Implications

This study demonstrates that an adaptive HRI driving system, aiming to maximize human–robot performance has to be carefully implemented. Indeed, this experiment highlights that the “human is still vital” [49], and is a key agent that needs to be taken into account when designing systems with operators in the control loop. For instance, a mixed-initiative interaction should switch the robot to manual control with ongoing high-scoring operators-like profiles to maintain their engagement and skills. Conversely, it should be preferred to trigger an autonomous mode with ongoing low-scoring operators-like profiles to prevent them from overloading. However, a decision system that drives human–robot interaction may not simply consist in regulating the level of automation. It is also critical to maintain the operators’ situation awareness when such mode transitions occur so that they are not only detected but also understood [43]; otherwise, conflict between the robot and the human could arise, therefore compromising mission success [49]. Several solutions have been put forward such as the use of cognitive countermeasures [50] and enhanced human–robot dialog [51] to improve automation transparency [52].

### 4.3. Future Research

As shown in Figure 7, Figure 8 and Figure 9, a high variability of ET and ECG features values is observed. This modulation is possibly related to the different events or different contexts during the mission. For instance, when leaks appear on the filling tank level it is normal to expect a high number of fixations on the related AOI. Thus, future research should consider the issue of how to aggregate the relevant features found with the mission context variables and automation level to better predict the mission performance. For instance it could be interesting to see how AOIs fixation pattern and HR/HRV features can relate with the external tank level, the number of leaks, the number of fires, the robot’s parameters and automation level. In particular, clustering algorithms could help identifying possible groups of features-context variables that would affect performance following the literature on cognitive states estimation. However, such a study would require a huge amount of data because of the need of sufficient representativeness (curse of dimensionality) appearing when many variables are considered. As in [34], a crowdsourcing platform can be exploited for this purpose.

Beyond that, and based on the achieved results of this paper, the next step in this research is to implement online methods to continuously infer the human operators’ performance like-profile (low versus high performer) and to launch adaptation based on the classification results obtained possibly used to feed a sequential decision-making process. More precisely, we believe that such an online performance profile prediction could be used to feed a stochastic sequential decision-making model dedicated to trigger adaptation [4,12].

Note that the conventional use of classifiers gives, in general, a one-shot label, performing an online tagging of the current input features. The certainty of this output label can be approximated by the accuracy of the classifier. For instance, different labels could be given as responses to similar features due to an inaccurate classification. It is known that classification errors can possibly bring the system to launch inappropriate actions. A promising solution to avoid launching inappropriate actions (e.g., actions based only on short term reasoning, called greedy actions) is to exploit the Partially Observable Markov Decision Process (POMDP) framework [4,12] to drive the human–robot interactions because: (i) it can integrate the uncertainties related to system states transitions (e.g., human actions) and related to observation functions (e.g., output labels from classifiers that are not always accurate); (ii) it can maintain a belief state distribution over possible human states (or operators-like profile); this belief state is updated each time an action is performed and an observation received using the Bayes rule; and (iii) its solution is an optimal policy—a function mapping reachable belief states to actions—that aims to maximize the expected long-term gains (i.e., entire system’s performance). In this sense, the belief state update process should estimate the system state (human, robot and mission states) during the interaction and choose to adapt the automation level of the robot with respect to the expected upcoming mission performance.

## Figures and Tables

**Table 1:** Average balanced accuracy results for 20×5-fold cross-validations and the 95% confidence interval of two-dimensional inter-subject single-trial classifications including different combinations of input features.

	Different Combinations of Input Features						
Classifiers	ECG, ET and Keys/Clicks	ECG and ET	ECG and Keys/Clicks	ET and Keys/Clicks	ET	ECG	Keys/Clicks
**kNN**	72%±3%	70%±4%	67%±3%	66%±4%	63%±3%	63%±3%	59%±3%
**LDA**	70%±3%	66%±3%	61%±3%	65%±3%	62%±3%	54%±3%	59%±3%
**QDA**	72%±3%	69%±3%	66%±3%	67%±3%	63%±3%	63%±3%	60%±3%
**SVM**	74%±3%	71%±3%	67%±3%	67%±3%	63%±3%	64%±3%	59%±3%
**GP**	74%±3%	72%±3%	68%±2%	68%±3%	65%±3%	64%±3%	60%±2%
**DT**	68%±4%	68%±4%	65%±4%	63%±4%	63%±4%	63%±4%	60%±2%
**RF**	72%±3%	70%±3%	65%±4%	65%±3%	62%±3%	64%±3%	60%±3%
**NN**	73%±3%	72%±3%	68%±3%	68%±4%	64%±3%	64%±3%	60%±3%
**ADA**	73%±3%	70%±3%	67%±3%	65%±2%	63%±3%	63%±3%	59%±3%
**NB**	69%±3%	68%±3%	65%±3%	62%±3%	60%±4%	62%±4%	50%±2%

**ECG features**: HR and HRV for 10-s time windows. **ET features**: total number of fixations in AOI1, in AOI2, in AOI3, in AOI4, and in AOI5 for 10-s time windows. **Keys/clicks features**: total number of keystrokes/clicks related to external tank task and to the robot navigation task for 10-s time window. Recalling all input features were conditioned on the automation level of the robot, which constitutes an additionnal common input feature for all tests. **kNN**: k-Nearest Neighbors; **LDA**: Linear Discriminant Analyses; **QDA**: Quadratic Discriminant Analyses; **SVM**: Support Vector Machine; **GP**: Gaussian Process; **DT**: Decision Trees; **RF**: Random Forest; **NN**:Neural Network; **ADA**: AdaBoost; and, **NB**: Gaussian Naive Bayes.
